# Quality, Usability, and Trust Challenges to Effective Data Use in the Deployment and Use of the Bangladesh Nutrition Information System Dashboard: Qualitative Study

**DOI:** 10.2196/48294

**Published:** 2024-09-30

**Authors:** Berhaun Fesshaye, Shivani Pandya, Lena Kan, Anna Kalbarczyk, Kelsey Alland, SM Mustafizur Rahman, Md. Mofijul Islam Bulbul, Piyali Mustaphi, Muhammad Abu Bakr Siddique, Md. Imtiaz Alam Tanim, Mridul Chowdhury, Tajkia Rumman, Alain B Labrique

**Affiliations:** 1 Department of International Health Johns Hopkins Bloomberg School of Public Health Baltimore, MD United States; 2 National Nutrition Services, Ministry of Health & Family Welfare Dhaka Bangladesh; 3 Nutrition Section, UNICEF Bangladesh Country Office Dhaka Bangladesh; 4 mPower Social Enterprises Ltd Dhaka Bangladesh; 5 Department of Digital Health & Innovation World Health Organization Geneva Switzerland

**Keywords:** digital health, nutrition, data for decision-making, health information systems, information system, information systems, LMIC, low- and middle-income countries, nutritional, dashboard, experience, experiences, interview, interviews, service, services, delivery, health care management

## Abstract

**Background:**

Evidence-based decision-making is essential to improve public health benefits and resources, especially in low- and middle-income countries (LMICs), but the mechanisms of its implementation remain less straightforward. The availability of high-quality, reliable, and sufficient data in LMICs can be challenging due to issues such as a lack of human resource capacity and weak digital infrastructure, among others. Health information systems (HISs) have been critical for aggregating and integrating health-related data from different sources to support evidence-based decision-making. Nutrition information systems (NISs), which are nutrition-focused HISs, collect and report on nutrition-related indicators to improve issues related to malnutrition and food security—and can assist in improving populations’ nutritional statuses and the integration of nutrition programming into routine health services. Data visualization tools (DVTs) such as dashboards have been recommended to support evidence-based decision-making, leveraging data from HISs or NISs. The use of such DVTs to support decision-making has largely been unexplored within LMIC contexts. In Bangladesh, the Mukto dashboard was developed to display and visualize nutrition-related performance indicators at the national and subnational levels. However, despite this effort, the current use of nutrition data to guide priorities and decisions remains relatively nascent and underused.

**Objective:**

The goal of this study is to better understand how Bangladesh’s NIS, including the Mukto dashboard, has been used and areas for improvement to facilitate its use for evidence-based decision-making toward ameliorating nutrition-related service delivery and the health status of communities in Bangladesh.

**Methods:**

Primary data collection was conducted through qualitative semistructured interviews with key policy-level stakeholders (n=24). Key informants were identified through purposive sampling and were asked questions about the experiences and challenges with the NIS and related nutrition dashboards.

**Results:**

Main themes such as trust, data usability, personal power, and data use for decision-making emerged from the data. Trust in both data collection and quality was lacking among many stakeholders. Poor data usability stemmed from unstandardized indicators, irregular data collection, and differences between rural and urban data. Insufficient personal power and staff training coupled with infrastructural challenges can negatively affect data at the input stage. While stakeholders understood and expressed the importance of evidence-based decision-making, ultimately, they noted that the data were not being used to their maximum potential.

**Conclusions:**

Leveraging DVTs can improve the use of data for evidence-based decision-making, but decision makers must trust that the data are believable, credible, timely, and responsive. The results support the significance of a tailored data ecosystem, which has not reached its full potential in Bangladesh. Recommendations to reach this potential include ensuring a clear intended user base and accountable stakeholders are present. Systems should also have the capacity to ensure data credibility and support ongoing personal power requirements.

## Introduction

### Background

Health information systems (HISs) play a critical role in aggregating and integrating data from health facilities and communities to improve health services’ delivery and health systems’ functioning [1]. However, the availability of high-quality, reliable, and sufficient data can be a challenge in low- and middle-income countries (LMICs), which are often characterized by fragmented governance structures, a lack of human resource capacity, and weak digital infrastructure and monitoring systems [2]. Nutrition HISs, also known as nutrition information systems (NISs), focuses specifically on nutrition-related indicators to understand and address challenges related to malnutrition and food security [3]. NISs can play a key role in shaping policy and programs for improving the nutritional statuses of populations and can guide nutrition programming integration into the routinely provided health services [3]. However, the bottlenecks that plague HISs broadly also impede the effective use of NISs. Disjointed efforts to improve the availability, accessibility, quality, and use of health data have limited countries’ ability to use data for health decision-making and problem-solving [4].

Evidence-based decision-making is integral to improving public health benefits and resources, and for translating quality evidence into action to achieve health impact, especially in LMICs [5-7]. Key components of adopting an evidence-based public health approach involve the systematic use of data and HISs, community engagement in decision-making, and having trust in the data being used for decision-making [8]. Although evidence-informed decision-making is strongly encouraged by key public health decision makers, the mechanisms of its implementation remain less straightforward and untested [7,9-13].

Data visualization tools (DVTs) have been recommended to support decision-making to better understand data and prioritize the next steps; however, their use remains an unexplored field of research in LMICs [10]. Dashboards, a type of DVT, are used to display relevant summary health performance metrics using visualization techniques (eg, graphs), which provide timely and actionable feedback that informs the decision-making of health providers (eg, health managers and supervisors) [11] and ultimately strengthens the overall quality of care delivered [14]. Dashboards enable the regular review of data by key stakeholders and decision makers in identifying data gaps and following up with corrective actions to improve overall health performance; moreover, they can capture information on data quality and completeness [2]. However, such as the use of HISs broadly—their existence alone is not a silver-bullet solution to improving data monitoring and increasing evidence-based decision-making. Furthermore, having more available data does not necessarily translate into more evidence-based programs and policies in global health [10]. Their impact on improving patient care and health outcomes remains unclear, with only a few studies having investigated how dashboards have been integrated into the decision-making process within primary health care systems [14].

### Need for Effective Data Use in Bangladesh to Track Nutrition Indicators

Bangladesh developed its NIS in the early 2010s, which is integrated into the national HIS. Bangladesh has one of the world’s largest deployments of the open-source District Health Information Software 2 (DHIS2) and has been able to integrate previously fragmented data systems into a central data repository—making Bangladesh one of the more digitally mature LMICs [15]. As Bangladesh’s nutritional programming is provided under 2 distinct directorates under the Ministry of Health and Family Welfare—the Directorate General of Health Services and the Directorate General of Family Planning—the NIS plays a critical role in integrating nutrition-related service data into 1 system which provides an overview of national nutrition progress [16]. The Nutrition Information and Planning Unit (NIPU)—a joint effort from the government of Bangladesh and UNICEF (United Nations Children's Fund)—was established to provide dedicated personnel to work with the NIS and support the use of its data for decision-making. Further, 1 of NIPU’s developments was the Mukto dashboard—which is a free, open-access platform to view nutrition-related performance indicators at the national, district, and subdistrict levels [16].

However, despite these efforts and advancements, the research agenda around the effective use of nutrition data to guide key priorities and decisions in Bangladesh remains relatively nascent. There is an increasing need to develop and strengthen the use of high-quality, reliable, and timely data to accelerate progress in achieving nutrition-related goals. This is especially of importance in Bangladesh, where—despite the substantial progress it has made in food access and nutritional status of the population—malnutrition and food insecurity continue to remain high: over 30% of children aged <5 years are stunted [17]; 22% of children aged <5 years are underweight [17]; over 50% of pregnant women are anemic [18]; and only around 35% of children aged 6-23 months are fed a minimum acceptable diet, highlighting inadequate infant and young child feeding practices in the country [17,18]. Addressing these nutritional needs has direct implications on both maternal and infant mortality as well as their quality of life.

### Objective of This Study

This study provides further context into the use of Bangladesh’s NIS, including the Mukto dashboard, and identifies areas of improvement so that the NIS can be used effectively and efficiently for evidence-based planning and data-driven actions to improve nutrition service delivery and, more broadly, the nutritional status of communities.

## Methods

### Study Setting and Context

Key informant interviews (KIIs) were conducted with stakeholders involved with both the NIS and the Mukto dashboard at the national level to understand the NIS landscape and challenges with data use. Participants were identified purposively through consultations with Bangladesh’s National Nutrition Services (NNS), UNICEF, and NIPU to identify those in both the government and nongovernment sectors that work with nutrition data and were perceived to use that data.

### Study Design and Data Collection

A total of 40 individuals were identified and contacted through email for interviews. Of those, 24 responded and were contacted via phone calls to confirm availability. Once confirmed, individuals were emailed once again with an invitation for participation and a brief description of study objectives. In total, 21 semistructured KIIs took place across 24 respondents. Further, 18 interviews were conducted individually and 3 were conducted in pairs. Paired individuals were from the same organization. Individuals interviewed were national stakeholders from both governmental organizations (n=12) and nongovernmental organizations (NGOs, n=12) including UNICEF, the World Health Organization, and the NNS, among others. Stakeholders were questioned on the perceived achievements of NIS, personal and organizational data sources for decision-making, as well as knowledge about Mukto and other nutrition dashboards.

Due to the COVID-19 pandemic, interviews were conducted virtually through Zoom (Zoom Video Communications, Qumu Corporation) between December 2020 and January 2021. All participants provided oral consent for both participation in the KIIs and recording of the interview. Semistructured interview guides created by study team members were used (Multimedia Appendix 1). KIIs were conducted by 2 facilitators (MIAT and TR), both of whom have postgraduate level training and received training on study design and research ethics. Further, 1 facilitator is the lead of mobile health initiatives at mPower, identifying as male, while the other is a research coordinator, identifying as female. Additionally, 1 facilitator led the KII and the second took notes with the 2 maintaining the same roles throughout each interview. KIIs ranged from 1 to 2 hours, were conducted in Bangla or English based on the interviewee’s preference, audio recorded, and subsequently transcribed and translated into English. Study team members based in Bangladesh (MIAT, MC, and TR) completed the transcriptions, translations, and verification of the KIIs. Upon review of initial interview transcripts, the study team determined that data saturation was reached, and additional interviews were not necessary.

### Analysis

**Figure 1:**
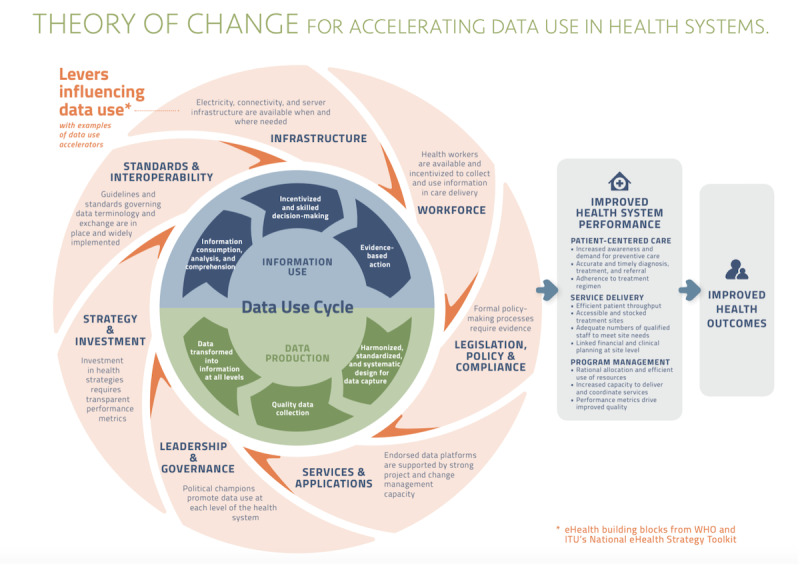
The PATH (Program for Appropriate Technology in Health) theory of change for accelerating data use in health systems. This theory explains how the data use cycle, made up of information use and data production, is influenced by levers such as infrastructure and workforce. These levers can act as accelerators for the data use process. All of these factors working together can contribute to improved health system performance, which in turn, leads to improved health outcomes. ITU: International Telecommunication Union; WHO: World Health Organization. Source: “Theory of Change for the Data Use Partnership [20] which is published under Creative Commons Attribution 4.0 International License [21] .

KII data were coded and analyzed using a thematic content analysis approach. Data analysis was conducted by a team of 4 (BF, LK, AK, and SP). An initial read-through of transcripts was performed to identify and discuss emerging themes. The creation of the codebook was guided by the PATH (Program for Appropriate Technology in Health) theory of change for accelerating data use in health systems (Figure 1; Multimedia Appendix 2) [19,20]. The theory of change framework was developed by PATH for visualizing the process of strengthening data systems and increasing data use, to improve health systems, for over 10 years. A formula that encapsulates the theory of change framework is “data production” plus “information use” multiplied by “levers and accelerators,” which leads to a culture of data use that allows for better health system performance [20]. Levers are the components such as workforce, leadership, governance, and infrastructure that set up an environment conducive to effective data use with accelerators increasing the impact of each lever. PATH has used the framework to create an investment roadmap for Tanzania to use data for improving the country’s health system and reach middle-income status [19]. The framework was chosen as a guide for qualitative analysis of the KIIs as it encapsulates the components of a robust data ecosystem. The codebook was iteratively developed to leverage the 7 components of the World Health Organization–International Telecommunication Union National eHealth Strategy within the PATH theory of change. The codebook was then adapted as the team of researchers read through the transcripts and identified emerging themes to include in the final codebook. Interviews were divided among the team for analysis, with illustrative quotes extracted for themes. Data were coded and managed in Excel (Microsoft Corp).

### Ethical Considerations

This study received approval from the Johns Hopkins University Bloomberg School of Public Health Institutional Review Board (14637) and received a nonhuman subjects research determination as defined by US Department of Health and Human Services regulations 45 Code of Federal Regulations 46.102. All participants provided oral consent before interviews and were allowed to opt out at any time. The presented data have been deidentified. No compensation was provided to participants.

## Results

### Overview

**Figure 2:**
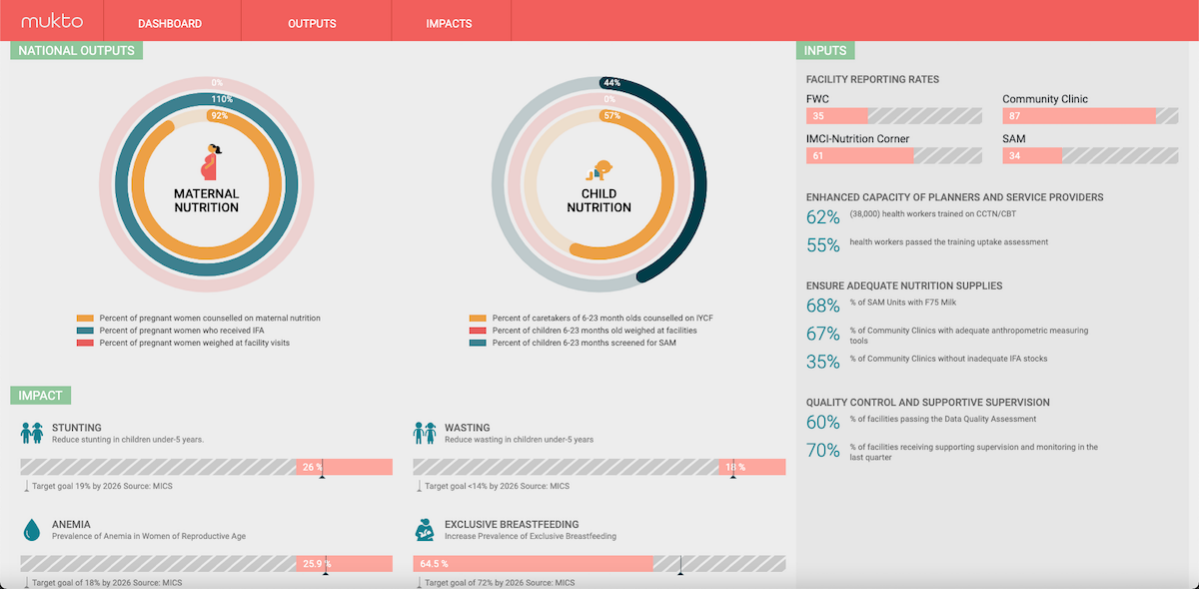
Nutritional Information System Mukto dashboard. A web-based platform for easy viewing of nutrition-related performance indicators at the national, district, and subdistrict levels in Bangladesh. FWC: Family welfare centre; IMCI: Integrated management of childhood illness; SAM:Severe acute malnutrition; CCTN: Comprehensive competency training on nutrition; CBT: Cash based transfers; IFA-Iron and Folic acid; IYCF -Infant and young child feeding; MICS-Multiple Indicator Cluster Survey; WHA-World Health Assembly; BDHS-Bangladesh Demographic and Health Survey.

The KIIs (n=24) provided important insights into how Bangladesh’s NIS is being used, including the Mukto dashboard (Figure 2 [22]), and the challenges that inhibited its use. Following an initial read-through of all interviews, high-level categories, known as levers, from the PATH framework were used to guide the creation of relevant subcategories. The predetermined levers include standards and interoperability, infrastructure, workforce, legislation, policy and guidance, services and applications, leadership and governance, and strategy and investment [19]. From these levers, this study team created subcategories that included indicator standardization, internet connectivity, and capacity development, among others. A summary of results, across all interviews, mapped according to PATH framework categories and our own subcategories are presented (Multimedia Appendix 3 [19]).

Additional cross-cutting themes emerged across these categories and subcategories including trust, data usability, personal power, and data use for decision-making. These themes provide further contextualization of the factors that support or inhibit the use of NIS by key stakeholders, and considerations for improvement to encourage its use and uptake.

### Trust

Trust was perceived as lacking in both the nutrition data sources and quality. Participants saw trust as an integral part of decision-making and without it, efforts at all levels—from data collected in the field to the policy level—contain faults. All interviewed stakeholders, except 1, commented on data quality and its shortcomings. Additionally, 3 government stakeholders and 5 NGO stakeholders expressed that data quality stems from the input stage and that any compromise there carries on throughout the lifecycle of the data. However, data input is not simply the responsibility of community health workers (CHWs), as clear and standard indicators must be well established beforehand. Manual processes for data input and management also affect quality as errors can go unnoticed.

The data that is coming from [the clinic level] is very weak, so if you think properly, you will see that the fault happened at the beginning.

NGO stakeholder

At the root, [data quality has been compromised] where the data is entered. Before that, the right indicators should be there. The design is important. If the design is faulty then every step after that will be faulty.

NGO stakeholder

Further, 2 government stakeholders stressed that data must be of high quality for use at the policy level. Without trust in where the data are coming from and what they comprise, there will be limited use of data to inform decision-making. Ambiguous and confusing data sources will only further diminish the credibility and assurance of the data ecosystem and impede data use and flow.

We just see the data, but to work on a policy level we have to be careful about the authenticity of the data, because you cannot provide misleading data on a national level.

Government stakeholder

However, 1 nongovernment stakeholder remarked that processes for data validation and avoiding duplicates require significant personal power especially with data coming from different sources, as is the case in Bangladesh. Although trust in data quality was lacking for many interviewees, 1 government stakeholder reported that their organization implements intensive data audits to ensure high-quality data:

There are monitoring officers…they monitor everything on their visit. They have a lot of mechanisms, and we visit them from the headquarter[s] to ensure quality…. we cross-check the data of the register with the MIS, and then the MIS with entered data. We cross-check the linkages among them. If there is any mistake, the links will not work. So everything is checked through a mechanism.

Government stakeholder

### Data Usability

Data usability was another issue identified by stakeholders. Indicators are not currently standardized across all organizations, regions, and data sources. For example, 1 nongovernment stakeholder stated that while their organization is aware of 64 standard indicators, they only focus on 25 of them. There is also a lack of standardization within health facilities and between government organizations. Gaps emerge in data collected if 1 health facility focuses on more indicators than another or if government organizations use inconsistent indicator definitions. Stakeholders noted that indicators should be standardized for maximum usability for decision-making, but this is a time-consuming process that requires clear leadership. Further, 1 NGO director noted that this leadership would ideally come from the government, but discussions around the standardization process have not yet occurred. Moreover, simply identifying indicators is not enough as there must be money and action to put those indicators to use.

Because it is not about just including indicators; a lot of money is needed to publish them as well. If two indicators are included in a list, it needs to be published, go to the facilities, and training should be provided again. Where will this money come from? So I would not say it is not harmonized at all. It was harmonized but it needs to be reviewed.

Nongovernment stakeholder

I remember [one] nutrition specific indicator, a small change was needed but we had to keep on telling them to change it for many days.

Nongovernment stakeholder

Another issue affecting data usability is the irregularity of data collection, which leads to an unclear picture of the country’s progress. Continuous data monitoring and review are critical to track the progress of data collection and implement iterative data quality improvements.

The problem is that [the data] is long term. One has to wait 4-5 years. In that case, if a sudden recommendation is required, then that old data has to be used from long ago, for example from 2014.

Government stakeholder

In many cases, I have observed that if we cannot follow up properly the data will not reach everywhere. So, you will find out that even after updating, a program is being carried out with the old data.

NGO stakeholder

Stakeholders also reported that there are usability differences between urban and rural data given that rural NISs are more established than urban systems. Health and nutrition efforts in Bangladesh have historically been focused on rural areas, leaving urban areas with inequitable access to these vital services [23]. Unlike rural areas, urban areas are structurally complicated, with less accountability and government support. While rural nutrition systems have been operating under the guidance of NNS and the Ministry of Health and Family Welfare, urban systems do not do so, with their reporting being voluntary and largely completed with the support of NGOs. Another discrepancy described between urban and rural areas is the lack of indicator standardization. Differences in rural and urban areas were a cause of concern for 13—over half (54%)—of the 24 interviewed stakeholders, from both the government and NGOs. Insufficient data negatively affects policy and investment decisions and diminishes the ability to understand the complete picture of the country’s nutrition status. Moreover, as Bangladesh experiences rapid urbanization, lacking sufficient urban data to improve health and nutrition could diminish national progress [23]. Further, 1 of the cited reasons for the lag in urban data is the dependence on NGO support without assistance from government entities and the lack of accountability that comes with this.

No one has told [urban areas] yet that the indicators are under the national system, and it is happening in rural, not in urban. It has to be done. It has not been communicated with the urban system on behalf of the government counterpart. So they don’t feel obliged to the nationally harmonized indicator-based reporting. So they only do it when someone tells them or backstopping them.

Nongovernment stakeholder

### Personal Power

Stakeholders reported that data collection, management, analysis, and monitoring are frequently executed manually, requiring significant personal power. Additionally, high staff turnover leads to the constant need for training. Reasons for staff turnover include transfer to other cadres, staff leaving after the completion of a project, and term-based NNS positions that do not set up a permanent workforce.

Many stakeholders also suggested that regular training of all relevant persons, such as CHWs, data analysts, and policy makers, should occur to help mitigate errors during data collection, management, and analysis. Currently, although there are trainers deployed at the field level and national levels—for government staff—there remain some gaps as government and NGOs variably provide such training to their respective staff. Further, 1 nongovernment stakeholder provided an example of a gap, which is that their organization provides less emphasis on nutrition training compared to other areas such as child health services and immunization.

Some technical systematic errors and [field workers] have to be motivated with one thing that the data entry is important. [Field workers] think of it as extra work. This thing, this reporting is often seen in a neglecting manner, NIS is not taken much seriously. I am doing a work, but if I do not report that work, it does [not] hold any value. Does it? I have to make a report. So I think we should put more stress and be aware of this reporting system and an information system above that. And those who work at the policy level, if they take this thing more seriously, then I think it is good.

Government stakeholder

But the way it has been presented, it seems that only you need the specialized kind of training or skill that only the NIPU team can do.

Nongovernment stakeholder

Infrastructural challenges, including poor internet connection at the field level, also exacerbate human resource issues. Internet issues were described as a barrier to both staff training and capacity development. Poor internet connection can interfere with data input, leading to poor quality or incorrect data reporting. Further, connectivity issues may affect staff workload and motivation.

However, still we face some problems with the network coverage, maybe he/she does not get the network coverage in office. But if he/she gets the network out of the office or at his home. For this issue, they take the register book with them at his home and work there. But he/she should not do this, but though he/she has the responsibility, he/she has to do it at any cost.

Government stakeholder

### Data for Decision-Making

The topic of data use for decision-making, and the ease of access to that data, was a central theme throughout the interviews. Stakeholders expressed the importance of evidence-based decisions and the necessity of timely and accurate data. Most participants reported receiving their data for decision-making directly from reports rather than from dashboards. These data were used to inform policy development, provide support, strengthen the understanding of data systems, and assess the impact data has on the government.

However, we use these data in many cases for example, for policy advocacy, trend analyzing, giving a better understanding to the government, showing them the difference, providing support to the places that have no support apart from additional supervision. We also use the data to observe if any government impact is being created for the input we are giving in the technical module and for the additional training that we provided.

Government stakeholder

The amount of data used for decision-making differs among stakeholders with some reporting that a vast amount of data is available, but not always being used. Available data sources also vary among organizations. For example, 2 government managerial stakeholders and 1 NGO director noted that their organization’s managers are aware of dashboards but do not use them as a primary data source. Further, 3 government stakeholders reported that data for decision-making is received directly through reports or their own personal networks, while 2 other government stakeholders receive their data from the national systems including the DHIS2, Directorate General of Family Planning Management Information System, and the Bangladesh Demographic and Health Survey 2014. Respondents expressed that data must be accurate for decision-making, which underscores the importance of data trust and data quality.

We can only observe the nutrition related indicators that have been collected on DHIS2 platform by MIS unit whereas they grant us access to those data. But we cannot [access] other nutrition indicators such as adolescent nutrition, elderly nutrition, or emergency nutrition on DHIS2 managed by MIS unit of the Ministry of Health.

Government stakeholder

If I say straightway about DHIS-2 that I want to see the information there about my division, district and upazila, the result will be shown there. I can get the information of four to five nutrition indicators which are very much important and to run a system or to see what is happening and what is the status of the nutrition in the country, it is easy to understand.

NGO stakeholder

Additionally, 1 government stakeholder noted that when making national-level decisions, the local context is not always considered. A nongovernment stakeholder stated that making decisions based on data at every level, such as the district and subdistrict, remains a challenge to be addressed. However, a different government stakeholder expressed that decision-making based on data does indeed occur at the subnational level.

I think these few achievements have helped to create a better process where a manager of either an event central level or upazila level can take some decisions based on the information.

Government stakeholder

A noted barrier to evidence-based decisions and dashboard use is personal power and staff motivation. Without sufficient human resources and incentives to use dashboards, the introduction of data dashboards may in turn increase the data and amount of work burden among existing CHWs.

So nutrition is one of the priorities of my activities and I will discuss about the dashboard with everyone and get solutions for it. If they provide us any decision regarding this, I think we can work so well from the national level. Especially managers, we send the program and data to them first and tell them about the situation. Then they consider it as a burden and think when will they do this pile of work.

Government stakeholder

Stakeholders were aware of health and nutrition dashboards, and while most did not use these for their decision-making process, some discussed the value of dashboards. Further, 1 government stakeholder expressed that dashboards allow for comparison between districts and facilities, which in turn facilitates decision-making and behavior change.

We almost get the national data from there [DGHS dashboard] or we can learn how other facilities of the health ministry are working. So it helps us to decide what we will do next and how we will do it. This sometimes helps in our decision-making.

Government stakeholder

## Discussion

### Principal Findings

This study sought to identify and understand how Bangladesh’s NIS can be improved to foster effective data use for increased coverage of nutrition services. Using PATH’s theory for accelerating data use (Figure 1), we organized our results to note gaps and areas for improvement and increase in data use for decision-making. The KIIs conducted with key government and NGO nutrition stakeholders identified several facilitators and challenges toward data use—largely centered around lack of trust in data, concerns around data usability, lack of personal power, and how that ultimately impacts data use for decision-making. We found that trust in the quality of nutrition data is lacking among stakeholders, mainly due to errors occurring during the data collection phase. These findings are similar to those conducted in Kenya and Malawi, which showed that routine data collected at the field level is regularly perceived as poor quality [24-26]. Possible reasons for poor quality data in other settings ranged from unstandardized data collection to lack of training [26], which echo the concerns of stakeholders in Bangladesh.

Perceptions of data usability were influenced by data quality and existing discrepancies between rural and urban data. Bangladesh’s subnational discrepancies in data have been perpetuated by the lack of accountability and support for urban areas coupled with a historic focus on nutrition services in rural areas [23]. Another usability issue noted is unstandardized nutrition indicators which lead to inconsistencies during data collection. Personal power was also a central theme as stakeholders reported that manual data management is hindered by high staff turnover and that staff training should take place regularly. Other studies in LMICs including Bangladesh, have also shown that field-level staff turnover is high while motivation is low, negatively affecting data quality and staff performance [27,28]. Poor internet connection at the field level is a barrier to personal power as it interferes with data collection, staff training, and capacity development. The importance of using data for decision-making was stressed by stakeholders, and despite knowledge of dashboards, such as Mukto, decision makers tend to receive data from their own preferred sources. A study conducted in Mozambique found that dashboards were viewed as an externally (nongovernment) developed strategy and garnered minimal interest from stakeholders, leading to the discontinuation of their use in this setting [25]. While our results did not reveal explicit reasons for the limited use of dashboards in the decision-making process, other settings may reveal possible causes. Addressing these possible causes will allow nutrition indicator dashboards, such as the Mukto dashboard, to reach their full potential.

### Data Ecosystems for Effective Decision-Making

Governments globally are increasingly using data to support their functions and decision-making capabilities [29]. Data systems are complex, and decision makers are often far removed from the entire data lifecycle—from data capture, compilation, distillation, and processing to visualization and reporting. While DVTs can be leveraged to improve the use of data for global health decision-making [10], we found that decision makers first have to trust that the data are believable, credible, timely, and responsive in order to use it. Generating this trust starts with having clear governance, leadership, and support for those who are at the genesis of the data ultimately presented in the summary program performance reports and dashboards. Trust also arises from relevant persons having salient roles in the construction and upkeep of the systems’ housing data [30]. These roles, contributing to a sense of ownership, are central to the success and longevity of the systems in place [30,31]. Ownership can also contribute to awareness of the data content, allowing for increased monitoring of the setting’s progress [30]. Continuous monitoring of progress and areas of improvement requires an ecosystem of reliable, timely, trusted, and actionable data that may arise from the use of digital visualization tools such as dashboards.

Importantly, data ecosystems should be localized and tailored to any setting or context and present key public health decision makers with actionable messages to optimize decision-making and translate knowledge into health practice, with the ultimate goal of improving health outcomes and reducing health inequalities. Ideally, all public health practitioners should incorporate evidence-informed decision-making during program implementation, policy development, and progress evaluation [12]. The data use ecosystem should foster the marriage between evidence-based public health interventions with real-world contexts and settings. The improvement in data quality and use will help facilitate the translation of findings into impactful policy recommendations and contribute to the improvement of overall health and well-being [8,32,33].

Results from this study indicate that the Bangladesh nutrition data ecosystem, which includes the NIS and the Mukto dashboard, has not yet reached its full potential. Gaps exist in nutrition data and decision makers do not feel confident in data collection and quality. Continued improvement in nutrition-related health outcomes will require tailoring of the Bangladesh nutrition data ecosystem along with increased ownership among stakeholders.

### Power, Trust, and Worker Motivation

Improving data quality, trust, and use cannot occur without establishing an environment that fosters worker motivation. CHWs, who are the cornerstone of primary health care systems globally by providing health services to hard-to-reach and rural communities, simultaneously play an important role in collecting health services (including nutrition-based services) data [27,34]. Bangladesh’s CHW program faces significant levels of attrition and high workloads, with some CHWs reporting discontent with their positions [27]. In our study, stakeholders expressed a lack of trust in data collected by CHWs and reported that field-level staff believed many tasks were extra work. However, the unrealistic workloads regularly placed on field staff [27] are likely the cause of the negative sentiments reported. Negative stakeholder perceptions of CHWs may be detrimental to the overall motivation and productivity of staff, further decreasing the quality of and trust in data. Motivation and satisfaction among CHWs can be undermined by both external factors such as program structure and provision of training, as well as internal factors such as satisfaction with work and CHWs’ perception of relevant stakeholders [28,34].

It is critical to also consider how gender and power dynamics impact relationships between field-level and high-level staff. Gender roles can shape CHW experiences and relationships with decision makers [35]. A majority of the global health workforce, which includes CHWs, are women with relatively low socioeconomic status and education, while only 30% of global health leadership roles are occupied by women [35,36]. Our interviewees expressed concern about the high worker turnover, yet specific reasons for this turnover were largely unspecified. Previous research has shown that reasons for attrition among women CHWs in Bangladesh include competing family commitments, disapproval from family members, and insufficient income [35,37]. Given Bangladesh’s reliance on CHWs for field-level data collection and service provision, a gender lens is critical for identifying appropriate solutions.

Some solutions that have been shown to increase health worker motivation include reasonable workloads, positive relationships with decision makers, sufficient monetary compensation, and recognition of efforts [27,28]. Without taking worker motivation and satisfaction into account, it is difficult to ensure high-quality data at the field level that decision makers will trust. Necessary improvements in worker motivation should come from those in leadership positions who have the power to affect change in work environments. Responsibility to increase data quality cannot fall solely on the backs of field-level workers as pressure to sustain large workloads and insufficient support from leadership are factors that decrease motivation and accuracy [27,28].

### Innovative Data Systems and System Improvements

The number of interactive dashboards and other DVTs, especially in the nutrition sector, has increased in recent years [38]. This is partly due to increased investments in nutrition that have increased the availability of data for decision makers and researchers [39]. However, the “build it and they will come” approach has been shown not to work [39] vis-a-vis health sector dashboards, leading to a larger discourse around better resource use and user-centered design of program monitoring systems [40]. We found that while government and nongovernment stakeholders were aware of dashboards, most did not use them in their decision-making process. Furthermore, the use of data dashboards does not guarantee improved data use and quality without sufficient user data literacy, important research considerations, and an established environment of trust among key decision makers. Moreover, the selection of key dashboard indicators to assess primary health care performance also depends on the availability of data, which often is lacking in LMICs [11]. Data ecosystems, which include data visualizations and dashboards, must have the capacity for constant improvements and iterative changes. Users must be motivated by the innovativeness and benefit of these tools to confidently and effectively use them in the decision-making process. Only with the effective integration of dashboards in health decision-making along with concurrent supporting policies, infrastructure, trust in data, empowered personnel, and an environment conducive to data use can the momentum of the data revolution be followed.

### Limitations

This study is not without limitations, Due to this study’s design, results may be influenced by social desirability bias. Additionally, due to the sample size and methods, results are likely not generalizable to other settings.

### Conclusion

We believe this study is valuable as it provides insights into stakeholder experience and suggestions for the effective use of nutrition data systems in Bangladesh. Although the creation of data systems and dashboards is important to increase the transparency of data, the mere creation of these systems may not solve Bangladesh’s NIS performance monitoring needs, as a clear intended user base, and accountable stakeholders must be present at the onset. Systems should be in place to ensure authenticity and credibility at the point of generation, with steps being taken to correct erroneous data. Data collection, management, and monitoring require robust, permanent human resources and strong infrastructure, as well as strong government and community engagement at each step of the data generation process.

Although these recommendations are not necessarily a prescription for success, increasing the presence of credible data ecosystems can enhance decision-making efforts. The culmination of many factors, such as appropriate infrastructure and trust, along with effective data use are necessary to keep up with the data revolution. While Bangladesh has made considerable progress in the nutrition field, ensuring that all future policy and governance decisions are informed by data is vital for continued improvement.

## Appendix

Multimedia Appendix 1 Semistructured key informant interview guide.

Multimedia Appendix 2 Qualitative codebook.

Multimedia Appendix 3 Contextualizing key informant interview results with the PATH (Program for Appropriate Technology in Health) framework.

## Abbreviations

CHW: community health worker
DGHS: Directorate General of Health Services
DHIS2: District Health Information Software 2
DVT: data visualization tool
HIS: health information system
KII: key informant interview
LMIC: low- and middle-income country
MIS: management information system
NGO: nongovernmental organization
NIPU: Nutrition Information and Planning Unit
NIS: nutrition information system
NNS: National Nutrition Services
PATH: Program for Appropriate Technology in Health
UNICEF: United Nations Children's Fund

## Data Availability

The data sets generated and analyzed during this study are available from the corresponding author upon reasonable request.

## References

[1] AbouZahr C, Boerma T (2005). Health information systems: the foundations of public health. Bull World Health Organ.

[2] Etamesor S, Ottih C, Salihu IN, Okpani AI (2018). Data for decision making: using a dashboard to strengthen routine immunisation in Nigeria. BMJ Glob Health.

[3] Salam RA, Das JK, Bhutta ZA (2019). Integrating nutrition into health systems: What the evidence advocates. Matern Child Nutr.

[4] Lippeveld T (2017). Routine health facility and community information systems: creating an information use culture. Glob Health Sci Pract.

[5] Aryeetey R, Holdsworth M, Taljaard C, Hounkpatin WA, Colecraft E, Lachat C, Nago E, Hailu T, Kolsteren P, Verstraeten R (2017). Evidence-informed decision making for nutrition: African experiences and way forward. Proc Nutr Soc.

[6] Majdzadeh R, Yazdizadeh B, Nedjat S, Gholami J, Ahghari S (2012). Strengthening evidence-based decision-making: is it possible without improving health system stewardship?. Health Policy Plan.

[7] van der Graaf P, Cheetham M, McCabe K, Rushmer R (2018). Localising and tailoring research evidence helps public health decision making. Health Info Libr J.

[8] Shafaghat T, Bastani P, Nasab MHI, Bahrami MA, Montazer MRA, Zarchi MKR, Edirippulige S (2022). A framework of evidence-based decision-making in health system management: a best-fit framework synthesis. Arch Public Health.

[9] Orton L, Lloyd-Williams F, Taylor-Robinson D, O'Flaherty M, Capewell S (2011). The use of research evidence in public health decision making processes: systematic review. PLoS One.

[10] Aung T, Niyeha D, Heidkamp R (2019). Leveraging data visualization to improve the use of data for global health decision-making. J Glob Health.

[11] Veillard Jeremy, Cowling Krycia, Bitton Asaf, Ratcliffe Hannah, Kimball Meredith, Barkley Shannon, Mercereau Laure, Wong Ethan, Taylor Chelsea, Hirschhorn Lisa R, Wang Hong (2017). Better measurement for performance improvement in low- and middle-income countries: the primary health care performance initiative (PHCPI) experience of conceptual framework development and indicator selection. Milbank Q.

[12] Brownson RC, Fielding JE, Maylahn CM (2009). Evidence-based public health: a fundamental concept for public health practice. Annu Rev Public Health.

[13] Bédard PO, Ouimet M (2016). Persistent misunderstandings about evidence-based (sorry: informed!) policy-making. Arch Public Health.

[14] Dowding D, Randell R, Gardner P, Fitzpatrick G, Dykes P, Favela J, Hamer S, Whitewood-Moores Z, Hardiker N, Borycki E, Currie L (2015). Dashboards for improving patient care: review of the literature. Int J Med Inform.

[15] Khan MAH, Cruz VO, Azad AK (2019). Bangladesh's digital health journey: reflections on a decade of quiet revolution. WHO South East Asia J Public Health.

[16] (2017). Guidance document on integration of priority nutrition indicators into existing systems and consolidation at national level. Bangladesh National Nutrition Council.

[17] (2020). Bangladesh demographic and health survey 2017-18. The DHS Program.

[18] (2021). Bangladesh: nutrition profile.

[19] Arenth B, Bennett A, Bernadotte C (2017). Definining and building a data use culture.

[20] (2016). Data use partnership: theory of change.

[21] Attribution 4.0 International (CC BY 4.0). Creative Commons.

[22] Mukto.

[23] Govindaraj R, Raju D, Secci F, Chowdhury S, Frere JJ (2018). Health and nutrition in urban Bangladesh: social determinants and health sector governance. Findings on urban health sector governance in Bangladesh.

[24] Admon AJ, Bazile J, Makungwa H, Chingoli MA, Hirschhorn LR, Peckarsky M, Rigodon J, Herce M, Chingoli F, Malani PN, Hedt-Gauthier BL (2013). Assessing and improving data quality from community health workers: a successful intervention in Neno, Malawi. Public Health Action.

[25] Gimbel S, Mwanza M, Nisingizwe MP, Michel C, Hirschhorn L, AHI PHIT Partnership Collaborative (2017). Improving data quality across 3 sub-Saharan African countries using the consolidated framework for implementation research (CFIR): results from the African health initiative. BMC Health Serv Res.

[26] Regeru R, Chikaphupha K, Bruce Kumar M, Otiso L, Taegtmeyer M (2020). 'Do you trust those data?'-a mixed-methods study assessing the quality of data reported by community health workers in Kenya and Malawi. Health Policy Plan.

[27] Roy S, Pandya S, Hossain MI, Abuya T, Warren CE, Mitra P, Rob U, Hossain S, Agarwal S (2021). Beyond institutionalization: planning for sustained investments in training, supervision, and support of community health worker programs in Bangladesh. Glob Health Sci Pract.

[28] Olaniran A, Madaj B, Bar-Zeev S, Banke-Thomas A, van den Broek N (2022). Factors influencing motivation and job satisfaction of community health workers in Africa and Asia-a multi-country study. Int J Health Plann Manage.

[29] Matheus R, Janssen M, Maheshwari D (2020). Data science empowering the public: data-driven dashboards for transparent and accountable decision-making in smart cities. Gov Inf Q.

[30] Watson-Grant S, Xiong K, Thomas JC (2017). Achieving sustainability in health information systems: a field tested measure of country ownership. Global Health.

[31] Watson-Grant S, Xiong K, Thomas JC (2016). Country ownership in international development: toward a working definition. Working Paper.

[32] Oxman AD, Lavis JN, Lewin S, Fretheim A (2009). Support tools for evidence-informed health policymaking (STP) 1: what is evidence-informed policymaking?. Health Res Policy Syst.

[33] Imani-Nasab MH, Yazdizadeh B, Salehi M, Seyedin H, Majdzadeh R (2017). Validity and reliability of the evidence utilisation in policymaking measurement tool (EUPMT). Health Res Policy Syst.

[34] Gottert A, McClair TL, Hossain S, Dakouo SP, Abuya T, Kirk K, Bellows B, Agarwal S, Kennedy S, Warren C, Sripad P (2021). Development and validation of a multi-dimensional scale to assess community health worker motivation. J Glob Health.

[35] Steege R, Taegtmeyer M, McCollum R, Hawkins K, Ormel H, Kok M, Rashid S, Otiso L, Sidat M, Chikaphupha K, Datiko DG, Ahmed R, Tolhurst R, Gomez W, Theobald S (2018). How do gender relations affect the working lives of close to community health service providers? empirical research, a review and conceptual framework. Soc Sci Med.

[36] Batson A, Gupta G, Barry M (2021). More women must lead in global health: a focus on strategies to empower women leaders and advance gender equality. Ann Glob Health.

[37] Alam K, Oliveras E (2014). Retention of female volunteer community health workers in Dhaka urban slums: a prospective cohort study. Hum Resour Health.

[38] Zhou B, Liang S, Monahan KM, Singh GM, Simpson RB, Reedy J, Zhang J, DeVane A, Cruz MS, Marshak A, Mozaffarian D, Wang D, Semenova I, Montoliu I, Prozorovscaia D, Naumova EN (2022). Food and nutrition systems dashboards: a systematic review. Adv Nutr.

[39] Manorat R, Becker L, Flory A (2019). Global data visualization tools to empower decision-making in nutrition. DNM.

[40] Labrique AB, Wadhwani C, Williams KA, Lamptey P, Hesp C, Luk R, Aerts A (2018). Best practices in scaling digital health in low and middle income countries. Global Health.

