# Supplementary material for: Quality, Usability, and Trust Challenges to Effective Data Use in the Deployment and Use of the Bangladesh Nutrition Information System Dashboard: Qualitative Study
Source: J Med Internet Res. 2024 Sep 30;26:e48294. doi: 10.2196/48294 (PMC11474113; doi:10.2196/48294)
Supplement: Multimedia Appendix 1 [file jmir_v26i1e48294_app1.docx]

**Assessment of Analysis of the Landscape of Nutritional Information System (NIS) in Bangladesh**

**Semi-Structured KII Guideline**

# **A.** **Respondent Basic Information**

| **Respondent Name:** | **Organization & Designation:** | **Working Area** |
| --- | --- | --- |
|  |  |  |

1. What are your responsibilities in relation to the following nutritional activity? Probe: Coordination, Monitoring, Data/Information Sharing, Report Preparation, Advocacy, Others
2. What nutrition-related indicators and information do you monitor regularly? Probe: PNRI Indicator, DLI Indicator, SAM, MAM.SAM, MAM, Complementary Feeding, EBF, IFA Supply and Distribution of Instruments, others.
3. How do you monitor and supervise the nutrition-related activities? Probe: From the nutrition-related report, Monthly meeting, visiting the facility, Nutrition Dashboard, others (Please mention).
4. Do you present any data and information at nutritional coordination meetings at the national level?
5. If yes, in which format do you present those data and information? Wherefrom do you get those data?
6. What kind of nutrition-related reports do you receive from your subordinate? And how often (e.g., monthly, quarterly, annually)?
7. Do you make any decision from the information you receive? Why or why not? Probe: Delay in getting data, erroneous data, inappropriate format, other.

# Input

1. How has the NIS (or nutrition-related activities, indicators, and data collection) changed the nutrition scenarios of the area/country in the last few years? Or Identify areas of increased accountability due to NIS

Probes:

- 1. Harmonization & Standardization of Nutrition Indicator across the institutions.
  2. Institutionalize use of data visualization tools at the district level
  3. Strengthen supportive supervision and routinize real-time monitoring and reporting
  4. Data-driven planning and performance tracking and accountability using scorecards and against set targets
  5. Strengthen the quality of data.

# Process

##

## **Assess the process for data quality control and monitoring:**

1. If you find any anomaly in the nutrition-related data, what are the steps you take for necessary corrections?
2. What parameters do you/your department use for data quality control? Probe: Whether data is collecting in a timely manner, whether collected data is complete, whether collected data is accurate, whether collected data is consistent, whether collected data is trustworthy
3. Who is responsible for data quality checking? What is the mechanism of data validation? Probe: Format check, Presence check, Range check, Type check, Consistent expressions (ex. Using one of St., Str, Street), Uniqueness (ex. Postal code)
4. What is the current process of data verification? Probe: Double-entry - entering data twice, Proofreading data - someone checks the data entered against the original document, Echo - system repeats the data being entered
5. Do you have any suggestions for improving the current system? Probe: Timeliness, Completeness, Trustiness regarding the Quality of Data

##

## Assess the availability and accessibility of nutrition-related dashboards and reports.

## Assess current usage of dashboards for day-to-day operational management and case tracking.

1. Do you use any information dashboard of nutrition for getting nutrition-related information.? Probe: DHIS2, HMIS, FP-MIS, Mukto, Other
2. What type of information are you looking for in the nutrition information system.
3. What are the benefits you get using the Nutrition information System? Probe: DHIS2, HMIS, MUKTO, Other

Probes: Do you provide any feedback to your subordinates based on the information you access from the NIS? If yes, how? If no, why not?

1. Do you need any additional information related to performing monitoring and supervision nutrition activities more effectively? Probe: Geolocation-wise malnutrition status, Facility wise activity performance, etc

Output

## Assesses Culture of data Usage

1. How does your organization currently use Nutrition data and information?
2. Do you allow/inspire your subordinates to make decisions/solve problems using the Information System? Why or why not? Probe: In all cases or few cases or not at all.
3. What changes are happening in data usage due to the nutritional information system?

Probe: Get own progress and performance in real-time, can export the data, get readily available reports, minimize the errors involved in manual record management e.g. duplication, make better decisions for operations, planning, supervision and monitoring.

**Achievements and Recommendation**

1. What significant changes have happened in the nutrition information system in the last couple of years? Probe: Infrastructure, reporting, capacity, information use, etc.
2. Do you have any suggestions/recommendations for improving the current nutrition information system?

**Probes:**

1. What are your thoughts about the Harmonization & Standardization of Nutrition Indicator across the institution?
2. What are your thoughts about sharing nutrition data with the public sector?
3. What are your suggestions to improve the quality of nutrition data?
4. What is your suggestion of individual capacity for data usage?
5. What is your suggestion for individual malnutrition child tracking?
6. What are your thoughts about community members should be involved in nutrition data usage?
