# Supplementary material for: Quality, Usability, and Trust Challenges to Effective Data Use in the Deployment and Use of the Bangladesh Nutrition Information System Dashboard: Qualitative Study
Source: J Med Internet Res. 2024 Sep 30;26:e48294. doi: 10.2196/48294 (PMC11474113; doi:10.2196/48294)
Supplement: Multimedia Appendix 2 [file jmir_v26i1e48294_app2.docx]

| ***Code Group*** | ***Code*** | ***Definition*** |
| --- | --- | --- |
| Standards & Interoperability | Indicator Standardization | Any discussion around standardized nutrition indicators so that the data being collected can be connected between different organizations and systems, or the lack thereof. |
|  | Interoperability | Any discussion of experiences and challenges with interoperability such as, for example, between NIS and other data sources such as DHIS-2, HIS, and nutrition counseling data. |
|  | Data validation | Any discussion of experiences with data validation or verification processes. |
|  | Data quality | Any discussion about the quality of data and approaches and challenges for improving data quality. |
|  | Rural vs urban differences in data | Any discussion around the differences in data accessibility between rural and urban, as well as the reasons for these differences. |
|  | Outdated data | Any discussion around outdated data – i.e., data that is within the information systems being greater than one year old. |
|  | Manual data collection/management | Any discussion of manual, or paper-based, data collection as it compares to automatic or digitized data collection (i.e., around human resources, reporting times to submit data, etc.) |
| Infrastructure | Internet Connectivity | Any discussion around the status of internet and network connectivity and how it impacts data reporting and data quality. |
| Workforce | Person Power & worker accuracy and motivation | Any discussion of the health workforce resourcing, availability, support, and their retention for collecting and reporting data. Accuracy of indicators during data collection and motivation from workforce to complete data input timely and accurately. |
|  | Need for training | Any explicit discussion around the need for initial and continued training for the health workforce. |
| Legislation, Policy & Guidance | Data use for decision-making and ease of access to data | Any explicit discussion which mentions the use of data for decision-making, including how this data is accessed, and which data sources are trusted for decision-making. Any explicit discussion of how easy or difficult it is to access the type of data needed for decision-making. |
| Services & Application | Capacity development | Any discussion around the level of capacity building occurring or needed to support all stakeholders who are involved in using the specified systems. |
| Leadership & Governance | Inter-ministerial involvement | Any discussion around inter-ministerial coordination or engagement in nutrition programs. |
| Strategy & Investment | Funding | Any discussion around how nutrition programs, information systems, workforce, etc. are being funded – and how they are being funded. |
|  | Data monitoring | Any discussion of how the government and other relevant stakeholders are monitoring and tracking nutritional data, including recommendations and challenges experienced. |
