# Supplementary material for: Quality, Usability, and Trust Challenges to Effective Data Use in the Deployment and Use of the Bangladesh Nutrition Information System Dashboard: Qualitative Study
Source: J Med Internet Res. 2024 Sep 30;26:e48294. doi: 10.2196/48294 (PMC11474113; doi:10.2196/48294)
Supplement: Multimedia Appendix 3 [file jmir_v26i1e48294_app3.docx]

| Category and subcategory | | Results from KIIs |
| --- | --- | --- |
| Standards and interoperability | | |
|  | Indicator standardization | While the importance of indicator standardization is understood, most nutrition indicators are not standardized, and the process of standardizing indicators is time-consuming. Rural and urban indicators are not the same and standards are not followed by all organizations. This was mentioned in 19 out of 21 interviews. |
|  | Interoperability | Interoperability between NIS^b^ and DHIS2^c^ should exist. Lack of interoperability increases personal power requirements. NIS should also be linked with HIS^d^ and nutrition counseling data. This was mentioned in 8 out of 21 interviews. |
|  | Data validation | Using validated data is essential, but the validation process is inadequate with data coming from multiple sources and gaps remaining unaddressed. Given that validation is a resource-intensive process, auto-validation could be a valid next step. This was mentioned in 10 out of 21 interviews. |
|  | Data quality | Poor data quality cannot be retrospectively improved. Poor quality data also inhibit decision-making. This was mentioned in 20 out of 21 interviews. |
|  | Rural versus urban differences in data | Rural data are much more accessible compared to urban data. Reasons for this include: urban areas are not under the jurisdiction of the Ministry of Health Planning, NNS^e^ does not provide similar support for urban areas compared to rural, and urban local governments regularly rely on NGO^f^ support. This was mentioned in 10 out of 21 interviews. |
|  | Outdated data | Follow-up and regular data collection are lacking and data that are used for decision-making are sometimes aged 3-5 years. It is difficult to make decisions and monitor progress when relying on outdated data. This was mentioned in 6 out of 21 interviews. |
|  | Manual data collection or management | Data collection is still mostly manual which increases personal power requirements and leaves room for error. This was mentioned in 11 out of 21 interviews. |
| Infrastructure | | |
|  | Internet connectivity | Poor internet connection is a barrier to manual data input at the health facility level, capacity building, and training. When community health workers (CHWs) do not have a proper internet connection, data input is delayed, and data quality is potentially compromised. This was mentioned in 7 out of 21 interviews. |
| Workforce | | |
|  | Personal power | Personal power is essential for data input, accuracy, analysis, and monitoring, but there is a high amount of staff turnover. Government NNS jobs are on a term system, so personal power is not permanent or guaranteed. Health workers experience high burdens due to inadequate staffing. This was mentioned in 13 out of 21 interviews. |
|  | Worker accuracy and motivation during data collection | Data accuracy is affected by worker motivation, inadequate internet connection, and lack of expertise. If data accuracy is compromised at the input step, this affects all future levels of data usage. This was mentioned in 10 out of 21 interviews. |
|  | Need for training | Since worker turnover is high, new employees must be trained, but refresher training should happen for CHWs, statisticians, MIS^g^ officers, and policymakers. However, frequent training requires high levels of coordination which is currently a barrier. This was mentioned in 14 out of 21 interviews. |
| Legislation, policy, and guidance | | |
|  | Data used for decision-making | For many, data are compiled into reports and used for decision-making, but data from dashboards is not being used for decision-making and policy formulation. Including local context in decisions is still a challenge. Data, when used effectively, can be harnessed for behavior change and increased performance. NIPU^h^ is currently playing a part in decision-making, but its sustainability should be ensured for future decision-making. This was mentioned in 19 out of 21 interviews. |
|  | Ease of access to data for decision-making | Access to indicators in DHIS2 is straightforward, but other indicators such as adolescent nutrition, older adult nutrition, or emergency nutrition are more difficult to access. Receiving data from the field level is also difficult at times due to late reports, which stem from infrastructure and workforce issues. While the functionality of data sources should be ensured, easy access to data does not guarantee its use during decision-making. This was mentioned in 14 out of 21 interviews. |
| Services and application | | |
|  | Capacity development | Capacity development is still an issue but is currently a work in progress. Data ownership is an important aspect of future capacity development as stakeholders feel as if they do not have control over the system they are using. This was mentioned in 14 out of 21 interviews. |
| Leadership and governance | | |
|  | Interministerial involvement | There is some coordination with ministries providing data and implementing nutrition programs, but that coordination could be improved. These interministerial issues will take time to address. This was mentioned in 9 out of 21 interviews. |
| Strategy and investment | | |
|  | Funding | Most funding is from the government and outside organizations such as UNICEF^i^. The use of these funds is an issue, for example, while nutrition may have sufficient funding, NIS does not. This was mentioned in 15 out of 21 interviews. |
|  | Data monitoring | While NIS has improved the monitoring process, there is still room for further improvement. Real-time monitoring is considered one of the first achievements of NIS. Improvements can be made in the digitization process and the creation of an NNS system to track nutrition-sensitive indicators outlined in Bangladesh’s NPAN2^j^. This was mentioned in 11 out of 21 interviews. |

^a^KII: key informant interview.

^b^NIS: Nutrition Information System.

^c^DHIS2: District Health Information Software 2.

^d^HIS: health information system.

^e^NNS: National Nutrition Services.

^f^NGO: nongovernment organization.

^g^MIS: management information system.

^h^NIPU: Nutrition Information and Planning Unit.

^i^UNICEF: United Nations Children's Fund.

^j^NPAN2: National Plan of Action for Nutrition 2.
